# Effects of *Clavispora* sp. co-fermented with *Saccharomyces cerevisiae* on physicochemical and aromatic profiles of blueberry wine

**DOI:** 10.3389/fmicb.2025.1708931

**Published:** 2026-01-12

**Authors:** Yan Ma, Zhi-qing Guo, Cheng Cheng, Jing Chen, Zhi-Peng Wang

**Affiliations:** 1 School of Marine Science and Engineering, Qingdao Agricultural University, Qingdao, Shandong, China; 2 Shandong Peanut Research Institute, Qingdao, Shandong, China; 3 Qingdao Zepha Agricultural Science and Technology Development Co., Ltd., Qingdao, Shandong, China

**Keywords:** blueberry wine, non-*Saccharomyces* yeast, sequential fermentation, anthocyanins, aroma profile

## Abstract

Blueberry wines provide a nutrient-rich alternative to perishable fresh fruits. However, the flavor complexity and nutrition retention remain limited by traditional fermentation. In this study, a non-*Saccharomyces* yeast *Clavispora* sp. LM32 was introduced into the *Saccharomyces cerevisiae* based blueberry fermentation. Ethanol levels were all reduced in simultaneous and sequential fermentation groups. Anthocyanin contents in simultaneous fermentation, sequential fermentation inoculating strain LM32 after 24 h (SEQ24), sequential fermentation inoculating strain LM32 after 48 h (SEQ48), were increased by 17.52, 38.59, and 43.87%, respectively. Meanwhile, SEQ24 brought significantly altered contents of volatile compounds, with higher alcohols increased by 19.85%, increased ethyl esters by 348.29%, acetate esters decreased by 18.43%, and synthesis of norisoprenoids and terpenes enhanced. Specially, contents of hexadecanoic acid ethyl ester and octanoic acid methyl ester in SEQ24 were increased by 4.6 times and 3.3 times, respectively, which can impart distinct tropical fruit and creamy fullness notes to the blueberry beverage. Sensory analysis demonstrated that sequential fermentation enhanced both fruity and sweet attributes. The study highlights the role of *Clavispora* sp. for enhancing aromatic profiles flavor and nutritional retention, proposing sequential fermentation as an optimal strategy for premium blueberry beverages.

## 1 Introduction

Blueberries (*Vaccinium corymbosum* L.) are nutrient-rich berries renowned for their health benefits. As consumer demand for nutritious foods rises, blueberries have gained significant popularity. They are widely consumed as fresh fruits, which however have a limited shelf life due to high perishability (Yuan et al., 2020). In response, fermenting blueberries into beverages becomes an effective solution to long-term storage and transportation (Yuan et al., 2018). Fermentation enhances the bioavailability of active ingredients in blueberries and introduces unique flavors and textures, making them increasingly appealing to consumers (Zhang et al., 2022).

In traditional brewing processes of fermented beverages, secondary aromas, which are the primary volatile components, originate from yeast metabolism during fermentation. However, fermentation employing *Saccharomyces cerevisiae* alone is inferior to spontaneous fermentation in diversifying the flavor of fermented beverages (Liu et al., 2016). As fruits are fermented, biochemical reactions can occur under the joint action of various microorganisms (Liu et al., 2022; Zhang et al., 2022). Recently, an increasing number of studies have shown that non-*Saccharomyces* yeasts play an active role in improving the quality of fermented beverages (Zhang et al., 2021; Vaštík et al., 2022; Morata et al., 2023; Klimczak et al., 2024).

Non-*Saccharomyces* yeasts are known for producing distinct aroma compounds, such as fruity esters, that contribute to a more diverse and appealing sensory profile compared to *S. cerevisiae* (Vaštík et al., 2022; Morata et al., 2023; Klimczak et al., 2024; Liu et al., 2022). Furthermore, they can help in regulating alcohol content and improve other beneficial components, like glycerol and polysaccharides, which affect the texture and mouthfeel of the fruit fermented beverages (Franco-Duarte et al., 2025). The objective of incorporating these yeasts in fermentation processes is to explore their metabolic characteristics and their ability to collaborate with *S. cerevisiae* to achieve a more complex, high-quality wine product. Currently, non-*Saccharomyces* yeasts have been used based on their potential to enhance the flavor complexity and quality of fruit fermented beverages. For example, species such as *Lachancea thermotolerans*, *Torulaspora delbrueckii*, and *Metschnikowia pulcherrima* have been studied for this purpose (Roudil et al., 2020). Additionally, mounting evidence highlights fundamental distinctions distinguishing non-*Saccharomyces* yeasts from conventional *S. cerevisiae* in synthesizing medium-chain fatty acids and higher alcohols. These differences may significantly alter ester concentrations in fermentation products (Tufariello et al., 2021).

In general, non-*Saccharomyces* yeasts specifically used for blueberry wine are lacking, which necessitates the exploration of new non-*Saccharomyces* yeast strains (Huang et al., 2022). Meanwhile, non-*Saccharomyces* yeasts in pure culture was detected to produce substances such as acetic acid, which are detrimental to the quality of blueberry wine (Englezos et al., 2022). To solve these problems while maintaining the positive effects or mitigating the negative ones of non-*Saccharomyces* yeasts on beverage quality, researchers have proposed strategies such as co-inoculation or sequential inoculation of non-*Saccharomyces* yeasts along with *S. cerevisiae* (Liu et al., 2022). Mixed fermentation approach is considered an effective way to optimize the fermentation process.

Among non-*Saccharomyces* yeasts, *Clavispora* sp. has stood out in the fermentation field. This species has been proven to enhance both the alcohol content and aroma complexity of tequila (Navarrete-Bolaños and Serrato-Joya, 2023). Additionally, when co-cultured with *S. cerevisiae*, *Clavispora* sp. also exhibits remarkable ester synthesis ability (Zhao et al., 2024). These characteristics suggest that *Clavispora* sp. may hold untapped value in blueberry fermentation. Meanwhile, it should be noted that the inoculation method (such as sequential inoculation or co-inoculation) and the nutritional composition of the fruit exert great influences on the production of aromatic compounds in mixed fermentation using non-*Saccharomyces* yeasts (Zhang et al., 2018). Therefore, further in-depth research should be carried out to understand the specific contribution of particular *Clavispora* sp. strains to the aroma of specific fruit-based fermented beverages.

This study aims to develop modified blueberry fermentation process by introducing the non-*Saccharomyces* yeast *Clavispora* sp. Simultaneous fermentation and sequential fermentation were evaluated for the physicochemical and aromatic profiles of blueberry wines. Meanwhile, the Hedonic tasting evaluation of blueberry wines by different fermentative modes were carried out. This study highlights the role of *Clavispora* sp. LM32 in improving flavor complexity and nutritional retention.

## 2 Materials and methods

### 2.1 Isolation and identification of yeast strains

Yeast strains were isolated from spontaneously fermented blueberry fruit (Zepha Blueberry Orchard, Jiaonan, China) using yeast extract peptone dextrose medium (YPD) agar (10 g/L yeast extract, 20 g/L peptone, 20 g/L glucose, 15 g/L agar) supplemented with 100 mg/L chloramphenicol to inhibit bacterial growth. After 48 h incubation at 25 °C, distinct colonies were purified by streak plating. ITS rDNA fragments were amplificated and sequenced by Tsingke Biotechnology (Beijing, China). The sequences were aligned in the NCBI database via BLASTn. Phylogenetic trees were constructed using MEGA11 (maximum-likelihood, 1,000 bootstrap replicates) (Varela et al., 2017).

### 2.2 Blueberry juice fermentation

The blueberry juice was placed into a sterile fermentation vessel (200 mL) made of glass. The initial composition of the juice was characterized by a sugar content of 80.46 g/L, a pH of 3.60, and a free amino nitrogen (FAN) concentration of 69.8 mg/L. Potassium metabisulfite (85 mg/L) was dissolved in the blueberry juice to inhibit spoilage microorganisms prior to fermentation. Subsequently, the fermentation vessel was transferred to a thermostatic incubator (25 °C). The following four methods were adopted for fermentation: commercial *S. cerevisiae* yeast (Angel, China) alone (AQ), simultaneous fermentation with the addition of both non-*Saccharomyces* yeast and *S. cerevisiae* (SIM), and sequential fermentation by adding non-*Saccharomyces* yeast strains first as the starter culture and then inoculating *Saccharomyces* yeast after 24 h (SEQ24) or 48 h (SEQ48). For simultaneous inoculation, both yeast strains were inoculated when their respective cultures reached 1 × 10^6^ cfu/mL, meaning the starting concentration of each strain in the must was 1 × 10^6^ cfu/mL. The fermentation progress was monitored by measuring the weight loss and tracking the pH changes. Fermentation was considered complete after 7 d, as this was the point at which weight loss and the pH stabilized, indicating that active fermentation had ceased. Post-fermentation aliquots were cryopreserved at −80 °C pending subsequent analytical procedures. Anthocyanins in fermented blueberry juice were determined by HPLC (Buckow et al., 2010).

### 2.3 Measurement of physicochemical properties of blueberry wines

The dried product after fermentation was used to substitute for soluble solids. Titratable acidity was determined by titration with 0.05 mol/L NaOH to an endpoint pH of 8.2. The pH value of the blueberry wines was measured using a pH meter (PB-10, Sartorius, Germany). Key analytical parameters including residual sugar, dry extracts, free SO_2_, total SO_2_, and ethanol in the blueberry wines was determined according to the previous study (Zhao et al., 2024; Zhang et al., 2018).

### 2.4 Extraction and identification of volatile organic compounds in blueberry wines

Volatile organic compounds were extracted via headspace solid-phase microextraction (HS-SPME) by Novogene (Beijing, China), and their composition and relative content were determined by gas chromatography–mass spectrometry (GC–MS). The method using HS-SPME as follows: First, add the sample to a 20 mL headspace bottle, cover and seal. Then, set the SPME condition: Use CTC three-in-one automatic sampler and an extraction fiber (DVB/Carbon WR/PDMS-80 μm). Extract material at 90 °C, shake for 5 min and extract for 20 min at the speed of 250 rpm. Resolution for 3 min. Set the GC cycle time to 41 min.

Analyses were carried out using a Thermo Fisher Trace1610-TSQ9610 (Thermo Fisher Technologies, Germany) system. DVB/Carbon-WR/PDMS-80 μm was used as a column. The carrier gas was high purity helium (>99.999%) at a constant flow rate of 1.0 mL/min. The temperature program of the oven was as followed: 50 °C as start temperature, 100 °C at a rate of 5 °C/min, 150 °C at a rate of 3 °C/min, 240 °C at a rate of 10 °C/min, hold on 2 min. The GC injector temperature was 240 °C. The MS Ion Source Temperature and ion source temperature were set at 240 °C and 280 °C, respectively. The mass spectrometer was operated in the EI mode at 70 eV using a range of m/z 40–400. Quantification was performed based on the peak areas of the standard compounds.

### 2.5 Hedonic tasting evaluation

The evaluation was made by 10 persons (4 females and 6 males, aged 23 to 38, with an average age of 28). Before evaluating the actual samples, all members underwent at least 15 h of training on aroma recognition and aroma intensity recognition (Tao et al., 2009). Beverage samples (15 mL) were served in ISO-standardized tasting glasses. All samples were labeled with a three-number code and presented to evaluators in randomized order.

The blueberry wines were evaluated in terms of sweet, floral, alcoholic, fruity, fermentative, and sour aromas by the evaluators using the scores ranging from 1 to 15 (1 = very low, 8 = moderate, 15 = very high). Five samples were evaluated per session, with each sample assessed twice in randomized order to ensure reproducibility.

### 2.6 Statistical analysis

All experiments were conducted with three independent biological replicates (*n* = 3). Data were analyzed using SPSS 26.0 (IBM Corp., Armonk, NY, United States) and presented as mean ± standard deviation. Significant differences among groups were determined by one-way analysis of variance followed by Duncan’s multiple range test (*p* < 0.05).

## 3 Results and discussion

### 3.1 Characterization of non-*Saccharomyces* yeast *Clavispora* sp.

**Figure 1:**
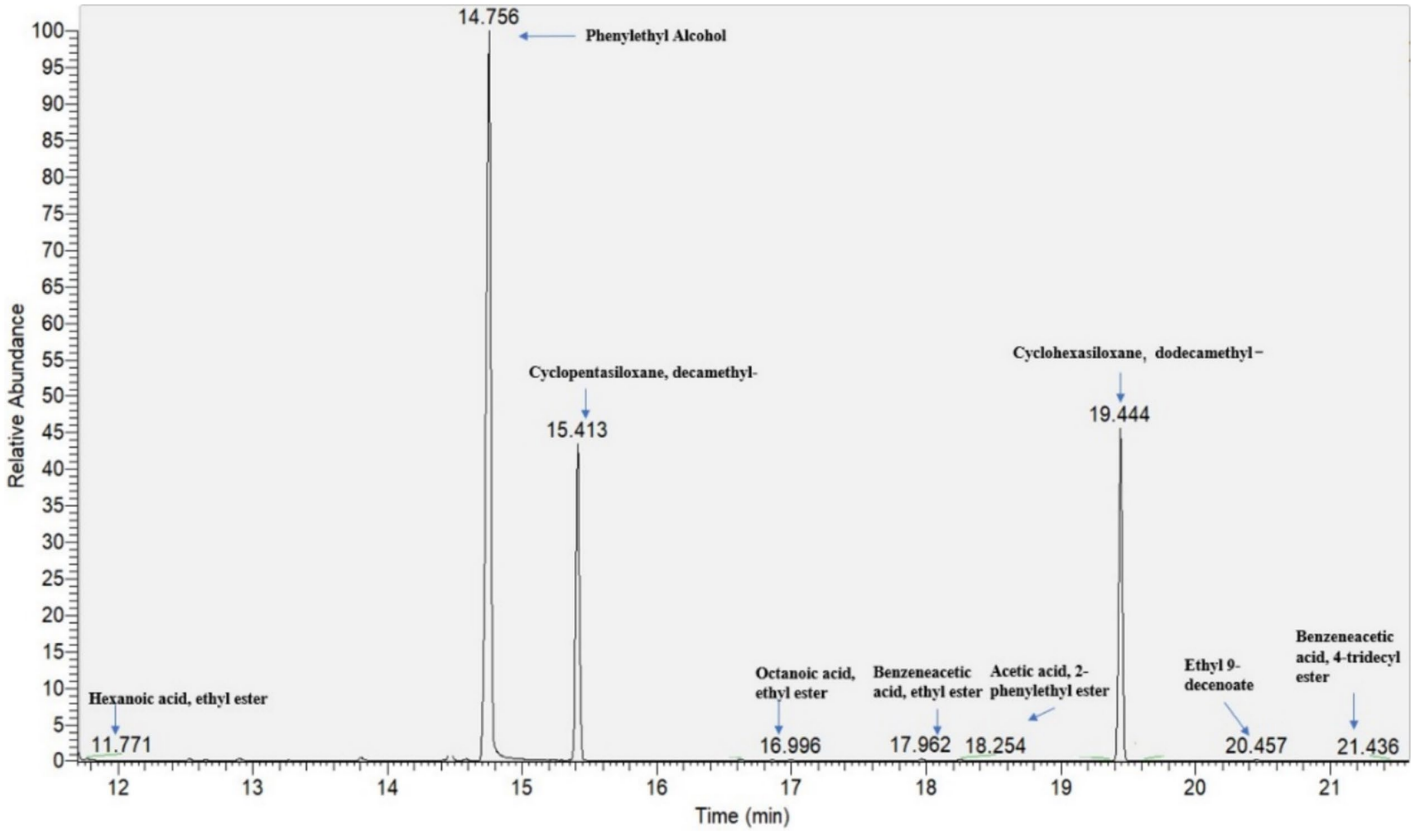
Volatile compound profile of strain *Clavispora* sp. LM32 in YPD liquid medium.

**Figure 2:**
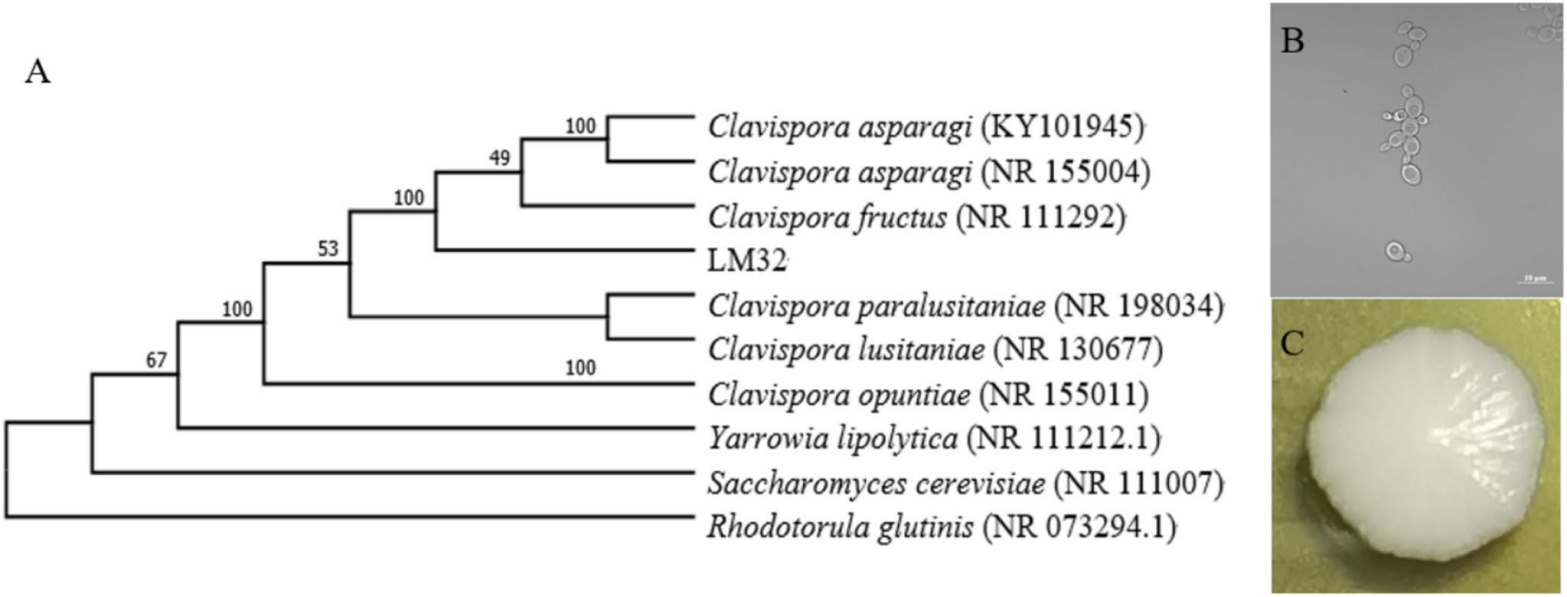
**(A)** Maximum-likelihood phylogenetic analysis of strain LM32 based on ITS sequences, Bootstrap values > 70%. **(B)** Confocal microscopy image of *Clavispora* sp. LM32 cells stained (63× oil immersion objective, scale bar = 10 μm). **(C)** Colony morphology on YPD.

From the spontaneously fermented blueberry fruit, yeast strains were selected and isolated by colony and cell morphology. Based on the Blastn results of ITS sequences, 33 strains were identified as non-*Saccharomyces* yeasts. Among these, strain LM32 in pure liquid culture was found to exhibit a superior fruity aroma compared to other strains, and was detected by GC–MS to produce significant esters and aromatic compounds (Figure 1). 2-Phenylethyl ester brings a characteristic floral and fruity aroma to the fermented product and is commonly used in fragrances to enhance scent experience. Benzeneacetic acid, ethyl ester, and ethyl hexanoate contribute fresh melon-like and fruity aromas, serving as the primary sources of the fruity characteristics (Liu et al., 2023). Additionally, medium- to long-chain fatty acid esters not only provide a creamy, rounded mouthfeel, but also interact synergistically with other aromatic compounds to make the overall flavor more harmonious (Fu et al., 2024). As shown in Figure 2, strain LM32 was obviously within the *Clavispora* clade of the phylogenetic tree, with similar colony and cell morphology as the *Clavispora* genus. Overall, the characteristics of strain *Clavispora* LM32 reveal its potential application value in fermentation to afford blueberry beverages.

### 3.2 Effects of co-fermentation on quality parameters of blueberry wines

To optimize blueberry fermentation, *Clavispora* sp. LM32 was co-inoculated with *Saccharomyces* yeasts as part of a co-fermentation strategy. As shown in Table 1, SIM resulted in the lowest residual sugar content and brought about ethanol levels comparable to those from AQ, whereas sequential inoculation produced higher content of residual sugar and significantly lower content of ethanol, with the SEQ48 showing a decrease of 10.9% in ethanol content. Notably, SEQ24 increased anthocyanin content by 43.8%, compared to AQ. As for titratable acidity, it remained stable across all fermentation groups.

**Table 1:** Basic physiochemical parameters of blueberry wines produced by different fermentation methods.

Parameters	AQ	SIM	SEQ24	SEQ48
Titratable acidity (g/L)	9.36 ± 0.20^a^	9.36 ± 0.18ᵃ	9.36 ± 0.15ᵃ	9.36 ± 0.22ᵃ
pH	3.35 ± 0.02ᵃ	3.35 ± 0.01ᵃ	3.34 ± 0.02ᵃ	3.32 ± 0.01ᵃ
Residual sugar (g/L)	9.15 ± 0.12ᵃ	8.46 ± 0.08ᵇ	9.65 ± 0.15ᶜ	9.25 ± 0.10ᵈ
Free SO_2_ (mg/L)	3.72 ± 0.15ᵃ	4.30 ± 0.12ᵇ	4.20 ± 0.10ᵇ	3.72 ± 0.15ᵃ
Total SO_2_ (mg/L)	20.49 ± 0.50ᵃ	17.38 ± 0.45ᵇ	17.38 ± 0.40ᵇ	17.38 ± 0.42ᵇ
Ethanol (% vol)	8.01 ± 0.05ᵃ	8.00 ± 0.04ᵃ	7.65 ± 0.06ᵇ	7.14 ± 0.07ᶜ
Anthocyanin (mg/L)	37.11 ± 1.05ᵃ	43.61 ± 1.20ᵇ	53.39 ± 1.50ᶜ	51.43 ± 1.30ᶜ

^a,b,c^ Values are reported as means ± standard errors (*n* = 3), different letters within a row indicate significant differences among treatments based on Duncan’s multiple range test at *p* < 0.05. AQ, pure inoculation of *S. cerevisiae*; SIM, co-inoculation of *Clavispora* sp. LM32 and *S. cerevisiae*; SEQ24 and SEQ48, sequential inoculation by adding *Clavispora* sp. LM32 as the start strain and then inoculating *S. cerevisiae* after 24 h and 48 h, respectively.

The lowest residual sugar content (8.46 g/L) was observed from the SIM, significantly lower than that of the AQ (9.15 g/L), which indicated that the synergistic effect between *Clavispora* sp. LM32 and *S. cerevisiae* may accelerate sugar metabolism (Table 1). In contrast, the SEQ24 saw the highest residual sugar content (9.65 g/L), which was probably ascribed to the lower sugar metabolism efficiency during the 24-h fermentation of *Clavispora* sp. LM32 alone, which resulted in a decreased sugar utilization rate by *S. cerevisiae* in subsequent fermentation. Compared to SEQ24, SEQ48 decreased the residual sugar content slightly (9.25 g/L), which however was still higher than that in the AQ. This suggested that extending fermentation time of *Clavispora* sp. LM32 alone only partially alleviated the inhibition of sugar metabolism.

In this study, sequential inoculation significantly lowered ethanol content compared to AQ (Table 1). In contrast, SIM resulted in an ethanol level (8.00%) comparable to that after AQ, which suggested that co-inoculation with *S. cerevisiae* suppressed *Clavispora* sp. LM32 growth (Pietrafesa et al., 2020). *Hanseniaspora guilliermondii*, another non-*Saccharomyces* yeast, in mixed fermentation systems reduced ethanol production by *S. cerevisiae* through preferential sugar consumption or generation of inhibitory metabolites such as acetic acid (Lombardi et al., 2018). Additionally, non-*Saccharomyces* yeasts may downregulate key enzymes in glycolysis, thereby reducing the accumulation of ethanol precursors and ultimately limiting the ethanol yield (Quirós et al., 2014). It has been reported that *Clavispora* species typically exhibit a strong ester-synthesizing capacity coupled with a low yield of ethanol from sugar, which consequently leads to reduced ethanol concentrations in mixed-culture fermentations. The low ethanol tolerance of non-Saccharomyces yeasts also contributes to the reduction of alcohol content in the fermentation broth (Fan et al., 2021; Rodríguez Machado et al., 2024).

All fermentation groups showed a titratable acidity of 9.36 g/L without significant differences (Table 1). This contrasts with the results reported in previous studies, *T. delbrueckii* significantly reduced the acid content (Wang et al., 2023). This was possibly due to that *Clavispora* sp. LM32 had a weaker metabolic regulatory ability for organic acids in blueberry beverages.

Much higher anthocyanin content was observed in the sequential inoculation groups compared with the AQ, with SEQ24 increasing the content by 43.8% (Table 1). Since the enhanced anthocyanin content was likely because non-*Saccharomyces* yeasts were capable of releasing more mannoproteins and polysaccharides, which reduced the adsorption of anthocyanins on the yeast cell wall, higher content of anthocyanins was retained in the fermented beverage (Tofalo et al., 2021; Zhang et al., 2021).

### 3.3 Effect of mixed fermentation on volatile compounds in blueberry wines

**Figure 3:**
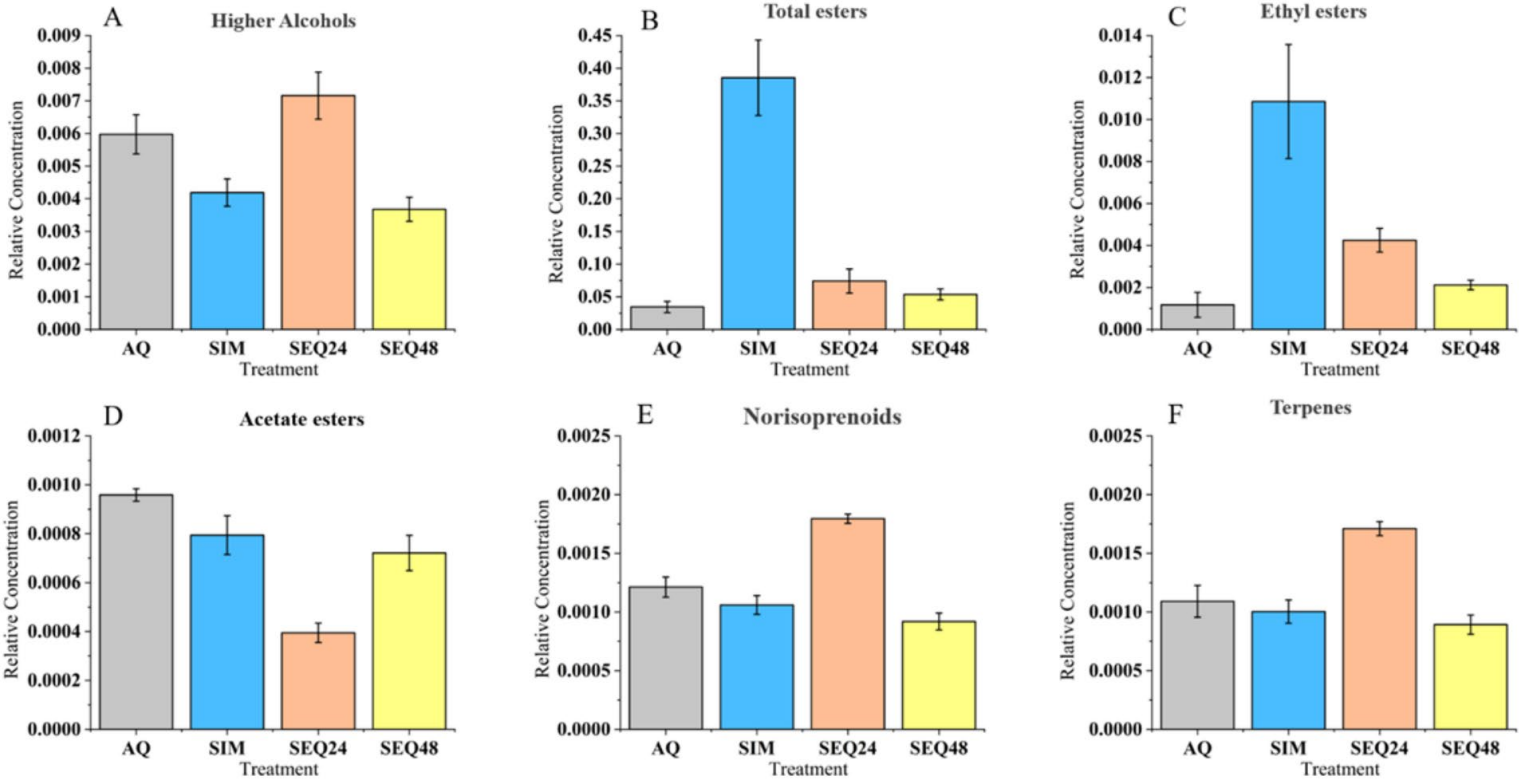
Effects of different fermentation methods on higher alcohols **(A)**, total esters **(B)**, ethyl esters **(C)**, acetate esters **(D)**, norisoprenoids **(E)**, and terpenes **(F)** in blueberry wines.

As shown in Figure 3A, SEQ24 resulted in increased relative content of higher alcohols by 19.85%, compared to AQ. In comparison, the SIM and SEQ48 fermented beverages had even lower content of higher alcohols. This result is similar to previous research, which used *T. delbrueckii* and *S. cerevisiae* in sequential fermentation (Loira et al., 2015). The study findings suggested that mixed fermentation using non-*Saccharomyces* yeasts and *S. cerevisiae*, particularly SEQ24, greatly enhanced the content level of higher alcohols in blueberry wines, thereby boosting the complexity of aromas, as well as improving the antioxidant activity of the beverage. It is speculated that the inoculation of non-*Saccharomyces* yeasts can alter regulatory mechanisms of amino acid catabolism in *S. cerevisiae*, influencing higher alcohol yield (Benito, 2018).

Esters primarily contribute to the fruity flavors in fermented beverages. Sequential fermentation greatly enhanced the relative content of total esters. The relative content of total esters in the SIM fermented beverage was almost 10 times higher than that in the AQ fermented beverage (Figure 3B). SEQ24 and SEQ48 showed an increase in the content by 1.5 and 1.2 times, respectively, compared to AQ. This can be explained by the increase in the relative content of ethyl esters (Figure 3C). Ethyl ester compounds mainly include fatty acid ethyl esters and branched-chain acid ethyl esters. Their biosynthesis begins with the reaction of substrate acid with coenzyme A under the catalysis of acyl-CoA synthetase to generate an active intermediate, acyl-CoA. Subsequently, in the yeast metabolic process, this intermediate undergoes esterification with ethanol through an alcoholysis reaction, ultimately forming ethyl ester compounds with characteristic aromas (Tondini et al., 2019).

On the contrary, sequential fermentation led to a reduce in the relative content of acetate esters in the fermented beverage, compared to AQ (Figure 3D). This was likely because the inoculation of *S. cerevisiae* inhibited *Clavispora* sp. LM32 to produce acetate esters. The involvement of *S. cerevisiae* regulated the production of acetate esters, which in turn had a positive effect on aroma (Liu et al., 2018). Therefore, the increase in ethyl ester content in the beverages from sequential fermentation suggested that the interaction between *S. cerevisiae* and *Clavispora* sp. LM32 has a synergistic effect on the production of ethyl esters while having an antagonistic effect on the production of acetate esters. It was speculated that SEQ24 enhanced ethyl ester synthesis by promoting the production of medium-chain fatty acyl-CoA by *Clavispora* sp. LM32. This provided abundant precursors for esterification with ethanol produced later by *S. cerevisiae*. Concurrently, the metabolic shift toward fatty acid biosynthesis diverted acetyl-CoA away from acetate ester formation (Fan et al., 2021; Rodríguez Machado et al., 2024).

Terpenes and norisoprenoids play a pivotal role in shaping the characteristic aromatic profile of blueberry wines, with their presence originating predominantly from the intrinsic biochemical composition of the blueberry fruit itself. In this study, the relative content of norisoprenoids in the SEQ24 was 48.05% higher than that in the AQ (Figures 3E,F). Terpenes and norisoprenoids are distributed in fruits as both free and glycosidically bound forms, with their glycosidic precursors being hydrolyzed during fermentation through either enzymatic or chemical pathways (Liu et al., 2019). The impact of mixed fermentation on terpene production may also be linked to nutrient balance. As mentioned earlier, nutrient competition in mixed culture suppresses the development of *Clavispora* sp. LM32, allowing more assimilable nitrogen to be utilized by *S. cerevisiae* (Del Fresno et al., 2021).

### 3.4 Analysis of aromatic compounds in blueberry wines

**Figure 4:**
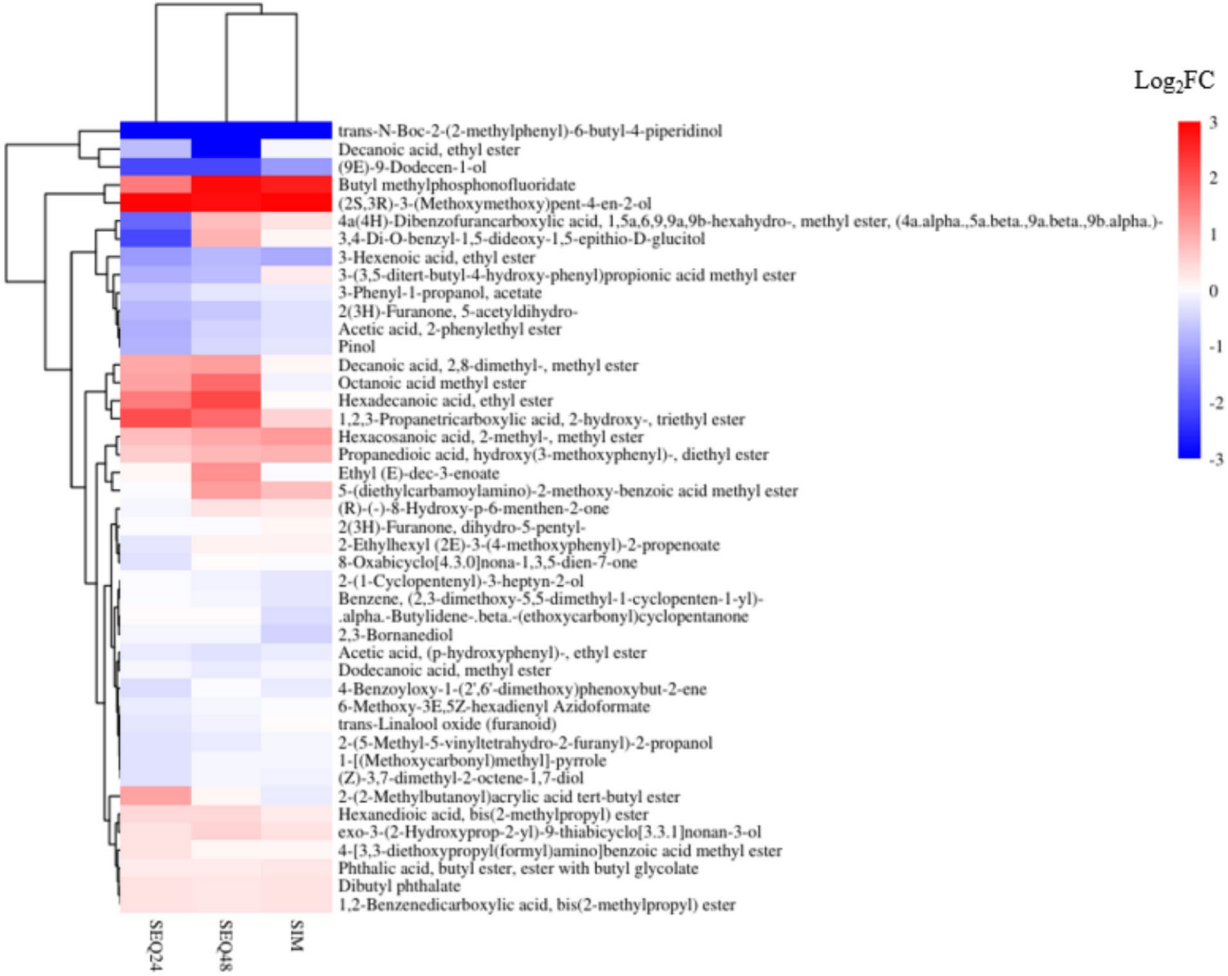
Heatmap representing log_2_-transformed fold changes of volatile compounds in simultaneous and sequential fermentation relative to pure *S. cerevisiae* fermentation.

The fold changes of volatile compounds analysis were analyzed to further demonstrate the difference of the three fermentation models. The study found that mixed fermentation using the non-*Saccharomyces* yeast *Clavispora* sp. LM32 and *S. cerevisiae* distinctly impacted the composition and content of ester compounds. As shown in Figure 4, SEQ48 increased the content of long-chain esters, especially ethyl palmitate (log_2_FC = 2.198) and methyl octanoate (log_2_FC = 1.735), which contributed prominent creamy and tropical fruity aromas to the beverage. The specific enrichment of long-chain esters revealed the regulatory effect of sequential fermentation on metabolic pathways. SEQ48 likely enabled *Clavispora* sp. LM32 to establish a unique metabolic steady state, resulting in differences in the expression of ester synthesis-related enzyme systems, compared to other fermentation methods (Zhang et al., 2024). Through the synergistic effect of triethyl propane-1,2,3-tricarboxylate (log_2_FC = 2.146) and *tert*-butyl 2-(2-methylbutanoyl)acrylate (log_2_FC = 1.108), SEQ24 significantly enhanced the complexity of flavor and caramel aroma while effectively reducing the content of undesirable volatile compounds like phenylethyl acetate (Minnaar et al., 2017).

**Figure 5:**
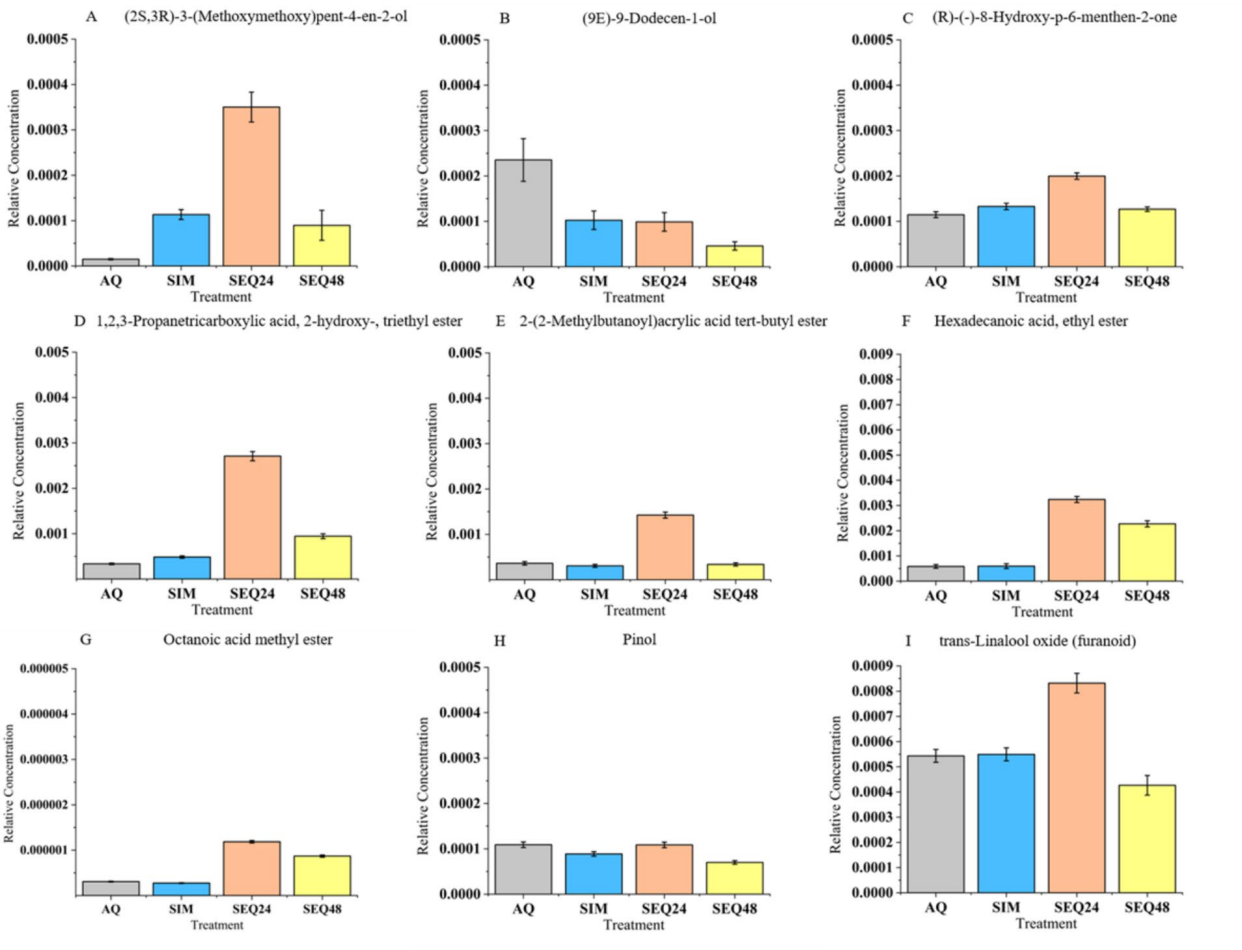
**(A–I)** Effects of different fermentation methods on marker volatile compounds identified.

In the regulation of higher alcohols, this study revealed the specific metabolic advantages of non-*Saccharomyces* yeast strains (Figure 5). SEQ48 significantly promoted the accumulation of (2*S*,3*R*)-3-(methoxy-methoxy)pent-4-en-2-ol (log_2_FC = 2.82), a compound with a fresh grassy plant aroma through which non-*Saccharomyces* yeasts enhanced the “grassy” sensory characteristics (Zhang et al., 2024). Notably, SEQ24 led to even stronger accumulation of this compound, which suggested that its metabolic flux might involve a unique redox enzyme system. This is similar to the mechanism of *Schizosaccharomyces pombe*, which regulates flavor precursors through the glycerol-3-phosphate pathway (Liu et al., 2018). Regarding off-flavor control, SEQ24 was more effective in reducing the content of unsaturated alcohol (9*E*)-9-dodecen-1-ol, alleviating potential fatty off-flavors. This finding expands current knowledge, suggesting that non-*Saccharomyces* yeasts not only reduce simple off-flavors like acetic acid, as previously reported, but also improve flavor balance by specifically degrading long-chain unsaturated alcohols (Liu et al., 2018).

In the regulation of terpenes and norisoprenoids, SEQ48 moderately reducing the content of pinol. This allowed the beverage to maintain a typical floral aroma while mitigating an overpowering resinous note. SEQ24 showed the most significant regulation of terpenes, among which (*R*)-(−)-8-hydroxy-p-6-menthen-2-one had stable content, while pinol reduced most. This selective retention may contribute to a fresher mint-floral complex aroma in the beverage. Notably, all three fermentation strategies maintained a stable level of 2,3-pinene diol, which confirmed that this compound originated from the blueberry matrix rather than fermentation metabolism (Varela and Borneman, 2017). Similar to findings in the literature on non-*Saccharomyces* yeasts (such as *M. pulcherrima* and *S. u*var*um*), the ratio regulation of esters, higher alcohols, and terpenes not only enhanced the complexity of the flavor but also significantly reduced the content of undesirable compounds (Liu et al., 2017). Notably, SEQ24 exhibited the best flavor balance, with its regulation pattern highly matching the yeast-matrix interaction effects observed in cold-climate grape varieties (Varela et al., 2016). This provides new insights for developing distinctive low-alcohol beverages.

In summary, the divergent changes of volatile compounds (Figures 4, 5) reflect species-specific metabolic pathways: the possible reason for this phenomenon is that *Clavispora* sp. LM32 preferentially synthesizes ethyl esters via acetyl-CoA transferase, while *S. cerevisiae* dominates acetate ester production. The sequential inoculation of SEQ24 may allow LM32 to establish metabolic fluxes toward fruity esters before ethanol inhibition occurs.

### 3.5 Hedonic tasting evaluation of blueberry wines

**Figure 6:**
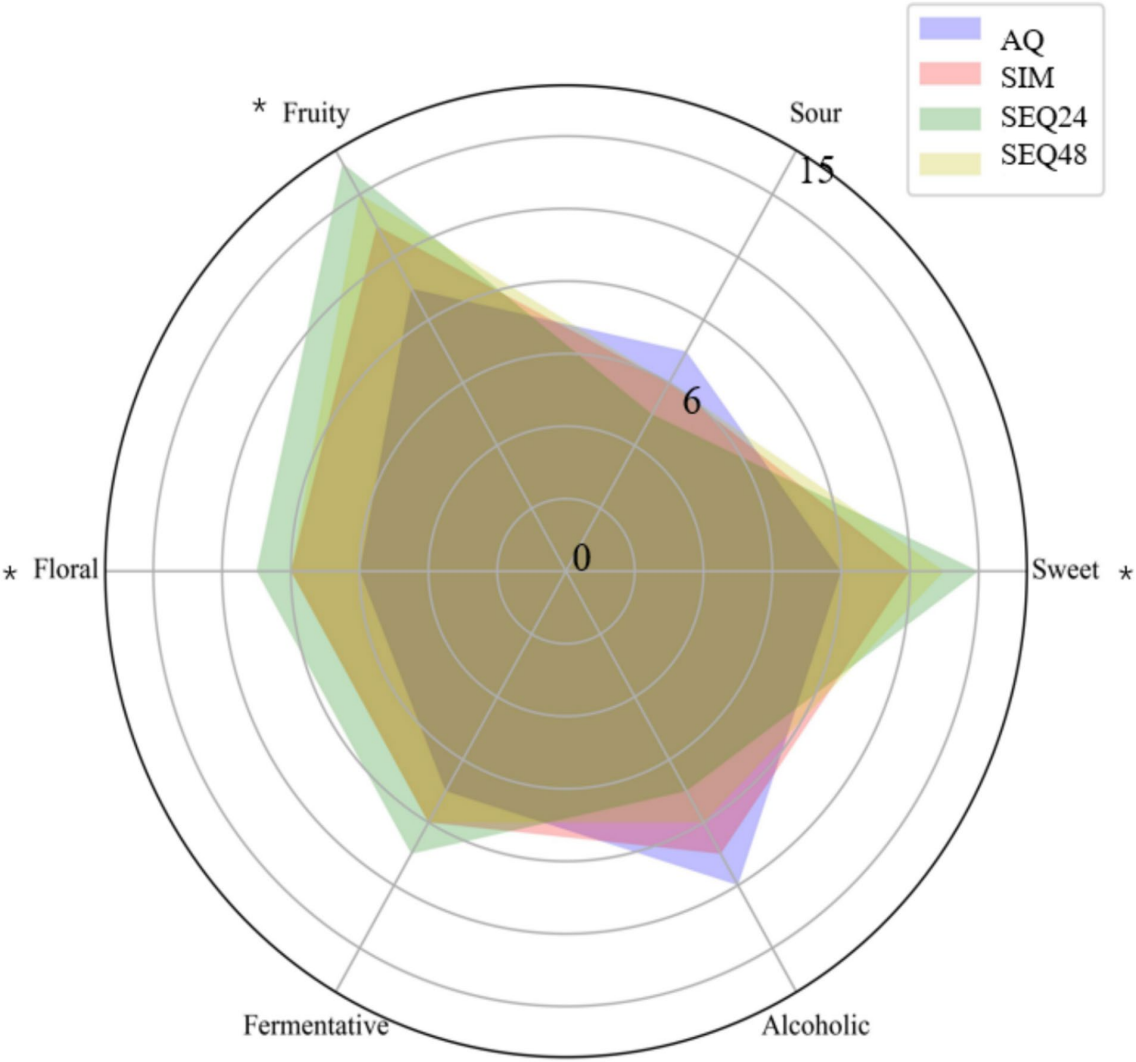
Sensory analysis of final blueberry wines. AQ, pure inoculation of *S. cerevisiae*y. Significance levels are analyzed by Duncan’s test as follows: *p < 0.05.

This study demonstrated that sequential inoculation with *Clavispora* sp. LM32 and *S. cerevisiae* (SEQ24) significantly optimized the sensory characteristics of blueberry wines. It enhanced fruity and floral aromas by promoting esterification reactions while reducing alcohol content and acidity and retaining certain natural blueberry traits. The AQ fermented beverage had a strong alcoholic flavor, and its intensity was significantly higher than those of the other beverages (Figure 6). Additionally, the sweet, floral, and fruity aromas of the AQ fermented beverage were notably weaker compared those of the beverages produced through mixed fermentation. The SEQ24 fermented beverage particularly exhibited the most intense sweet, floral, and fruity aromas. Moreover, sequential fermentation, especially SEQ24, reduced the acidity and alcohol content of the blueberry wine. The reduction in acidity in the SEQ24 fermented beverage was likely because *Clavispora* sp. LM32 reduced acetate production through metabolic diversion during the separate fermentation phase, promoting esterification reactions and enhancing its fruity aroma. Compared to *M. pulcherrima* strains used in a previous study to ferment Rosé wines, *Clavispora* sp. LM32 in SEQ24 demonstrated a stronger ester synthesis capability while maintaining the natural fruit acid characteristics (Muñoz-Redondo et al., 2021). This characteristic made it more suitable for developing low-alcohol, intensely-fruity functional blueberry beverages, catering to the dual demands of modern consumers for health and flavor. Future research should involve consumer acceptance tests to validate the actual effects of sensory optimization and explore the compatibility of *Clavispora* sp. LM32 with different yeast strains to expand its application range.

## 4 Conclusion

This study delved into the effects of *Clavispora* sp. on physicochemical and aromatic profiles of blueberry wine. The SEQ24 sequential fermentation strategy offers significant advantages with low alcohol, intense fruity aroma, and high functional compound content. The study highlighted the role of *Clavispora* sp. for enhancing the quality of blueberry wine. The strategy holds potential value for application to fruit-based fermentation utilizing fruits other than blueberry.

## Data Availability

The raw data supporting the conclusions of this article will be made available by the authors, without undue reservation.
